# The Main (Glyco) Phospholipid (MPL) of *Thermoplasma acidophilum*

**DOI:** 10.3390/ijms20205217

**Published:** 2019-10-21

**Authors:** Hans-Joachim Freisleben

**Affiliations:** 1 Goethe-Universität, Gustav-Embden-Zentrum, Laboratory of Microbiological Chemistry, Theodor-Stern-Kai 7, D-60590 Frankfurt am Main, Germany; hj.freisleben@t-online.de; 2 Universitas Indonesia, Medical Research Unit, Faculty of Medicine, Jalan Salemba Raya 6, Jakarta 10430, Indonesia

**Keywords:** *Thermoplasma acidophilum*, *Thermoplasma volcanium*, tetraether lipid, main phospholipid, black lipid membrane, Langmuir-Blodgett monolayer, liposomes, proton permeability, bacteriorhodopsin reconstitution, light-driven ATP synthesis

## Abstract

The main phospholipid (MPL) of *Thermoplasma acidophilum* DSM 1728 was isolated, purified and physico-chemically characterized by differential scanning calorimetry (DSC)/differential thermal analysis (DTA) for its thermotropic behavior, alone and in mixtures with other lipids, cholesterol, hydrophobic peptides and pore-forming ionophores. Model membranes from MPL were investigated; black lipid membrane, Langmuir-Blodgett monolayer, and liposomes. Laboratory results were compared to computer simulation. MPL forms stable and resistant liposomes with highly proton-impermeable membrane and mixes at certain degree with common bilayer-forming lipids. Monomeric bacteriorhodopsin and ATP synthase from *Micrococcus luteus* were co-reconstituted and light-driven ATP synthesis measured. This review reports about almost four decades of research on *Thermoplasma* membrane and its MPL as well as transfer of this research to *Thermoplasma* species recently isolated from Indonesian volcanoes.

## 1. Introduction

The scientific history of *Thermoplasma* species started in 1970, when Darland and co-workers isolated a “thermophilic, acidophilic mycoplasm” from a self-heating coal refuse pile [1]. *Thermoplasma acidophilum* generated its own micro-environment of sulfuric acid at pH 1–2 and 55–56 °C by oxidative degradation of pyrite-containing material and was grouped into the genus *Mycoplasma* because it lacks a cell wall. Hence, the cytoplasmic membrane must be sufficiently resistant towards the extreme environmental milieu by its unique membrane components, mainly tetraether lipid (TEL) [2]. It took some more years, until Archaebacteria were introduced [3], the phylogeny of the mycoplasmas re-analyzed [4], and *Thermoplasma acidophilum* re-grouped into the Archaeal order of *Thermoplasmatales,* family *Thermoplasmataceae*, where it was first the only species, later accompanied by *T. volcanium* [5].

Physico-chemical characterization of the membrane lipids of *Thermoplasma* (as reported here) was mainly performed at the Goethe-University Frankfurt am Main with a laboratory stem DSM 1728 Göttingen, Germany. This research was transferred to Indonesia [6,7,8], since *Thermoplasma* and other Archaeal species had been found in Indonesian volcanoes [9]. Meanwhile, Archaea have been isolated and cultured from there [10,11] and research will be continued from wild type species, until Indonesian laboratory stems will have been established.

## 2. Growth of *Thermoplasma*

*Thermoplasma acidophilum* (*T.a.*) DSM 1728 Göttingen, Germany, or isolated from the Indonesian volcano Tangkuban Perahu was cultured micro-aerobically in 1- or 2-L flasks on a shaker [11] or in 5-, 10- or 50-L fermenters according to [12] at 39–59 °C and pH 1–2 in Freundt’s medium containing sulfuric acid adjusted to the desired pH.

Optimal fermenter growth occurs at pH 2; more selectively—although slightly slower—*Thermoplasma* can be grown in culture at pH 1.5, because the conditions under pH of 2 and 60 °C are quite resistant towards contamination with other microorganisms. *Thermoplasma* cells are pleomorphic ranging in size between 0.5 and 5 µm with a maximum of size distribution at 2–3 µm [12]. EPR measurements estimated an average inner volume of 1 µm^3^ and an outer diameter of 2 µm (estimations under the presumption of spherical shape) [12,13,14,15].

**Figure 1:**
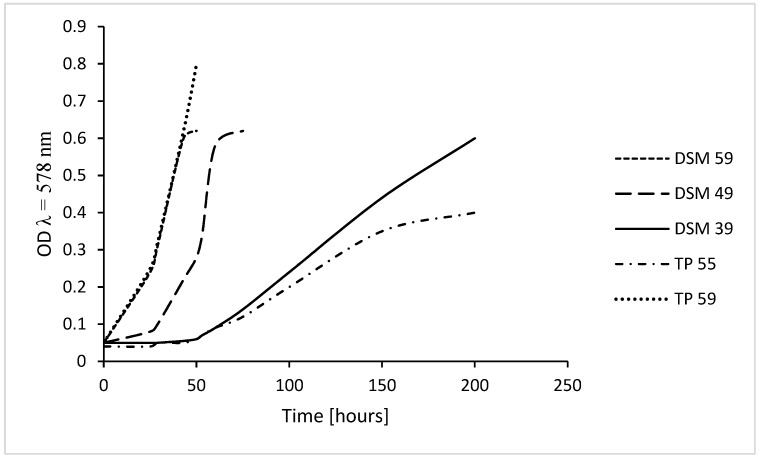
Culture growth of *Thermoplasma*. Growth of *Thermoplasma acidophilum* DSM 1728, Göttingen, Germany, at 39 °C, 49 °C, and 59 °C and of wild type *Thermoplasma* harvested from a Tangkuban Perahu crater, grown at 55–56 °C in 1-Liter culture flasks without automated control (TP 55); growth of the latter is similar to that of the former at 39 °C to a maximum OD of 0.35–0.4 and at 59 °C in a computer-regulated 2-L fermenter (TP 59); OD, optical density at λ = 578 nm; in all cultures, pH was below 2 (at the beginning of the cultures adjusted to 1.5 and re-adjusted, if necessary); values adapted from [11,12].

*Thermoplasma acidophilum* DSM 1728 grows up to OD (λ = 578 nm) of 0.6, at 39 °C within 200–210 h, at 49 °C within 70 h, at 59 °C within 42 h [12], wild type from Tangkuban Perahu, Indonesia, identified as mixtures of *T. acidophilum* and *volcanium* [11], grows up to OD of 0.35 within 160 h in culture flasks on a shaker without automated regulation of growth conditions at 55–56 °C in 1–5 sequential culture generations and up to OD 0.4 within 240 h [11]. In an optimized computer-regulated fermenter culture wild type grew up rapidly to an OD of almost 1.0 within 2-3 days (Figure 1); this culture has not yet been further investigated. 

**Figure 2:**
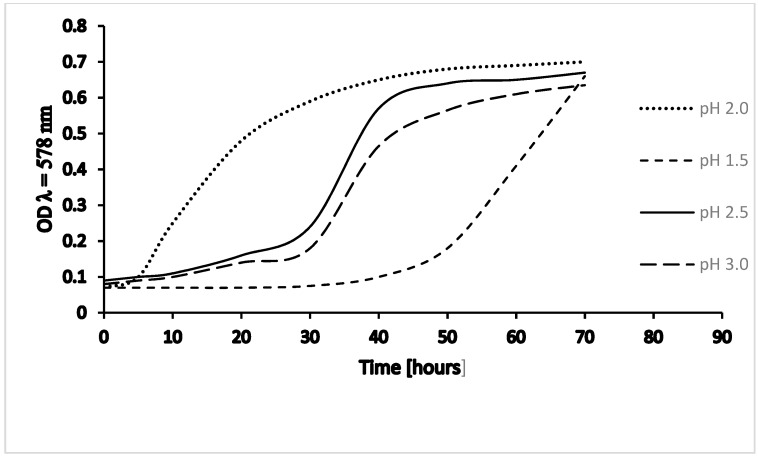
Culture growth of *Thermoplasma acidophilum* – pH dependency. Growth of *T.a*. DSM 1728, Göttingen, at 59 °C and varying pH; OD, optical density at λ = 578 nm; values adapted from [12].

Culture growth at various pH (1.5; 2.0; 2.5; 3.0) demonstrated that growth optimum under laboratory conditions is at pH 2. Growth curves at pH 2.5 and 3.0 are similar, but growth is slower than at pH 2.0; and at pH 1.5, growth is considerably slower and shows a different kinetic curve in the range of our measurements (Figure 2). 

## 3. Cell Lysis Experiments

Lysis experiments with *T.a*. cells under various conditions (e.g., different lysis buffers at lysis temperature of 22 or 59 °C) had shown clear maximal resistance of the *T.a*. cell membrane at pH 4 [16,17].

**Figure 3:**
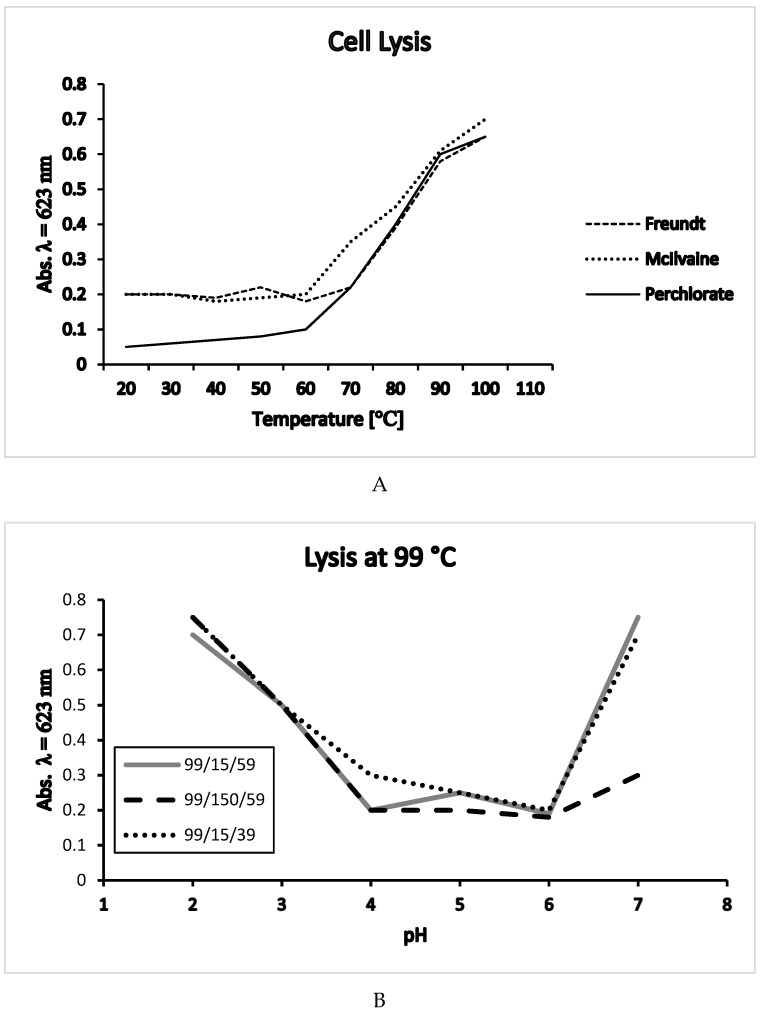
(**A**) Lysis experiments with *Thermoplasma acidophilum* cells in different buffers. (**B**) Lysis experiments with *Thermoplasma acidophilum* cells at 99 °C; 99/15/59; 99 = lysis temperature, 99 °C/15 = dilution with 15 mM sodium chloride solution/growth temperature of cells, 59 °C; 99/150/59; 99 = lysis temperature, 99 °C/15 = dilution with 150 mM sodium chloride solution/growth temperature of cells, 59 °C; 99/15/39; 99 = lysis temperature, 99 °C/15 = dilution with 15 mM sodium chloride solution/growth temperature of cells, 39 °C; adapted from [16].

The influence of temperature on membrane stability in different media, Freundt’s solution, McIlvaine buffer and after protein precipitation with HClO_4_ is shown in Figure 3A. In McIlvaine buffer cells begin to lyse at 60 °C, immediately above their growth optimum of 59 °C. In Freundt’s solution cells begin to lyse at 70 °C, similarly after protein precipitation with HClO_4_, only in the latter case absorbance starts—as expected—at a lower level [16].

Cell lysis at 99 °C, after dilution of McIlvaine lysis buffer with 15 mM and 150 mM sodium chloride solution is shown in Figure 3B. Cells were grown at 39 or 59 °C. Highest membrane stability can be seen between pH 4 and 6. At growth optimum of pH 2, the membrane appears less stable. 

The picture in Figure 3B differs from lysis conditions with the same lysis buffers, but at incubation temperatures of 22 and 59 °C. These conditions reveal a clear maximum of membrane stability at pH 4 [16,17]. Lysis is influenced by both, growth temperature and lysis temperature; at incubation temperature higher than 60 °C membrane stability rapidly decreases under pH 2 and over pH 5. At growth temperature of 39 °C cells lyse more rapidly than cells grown at 59 °C. 

## 4. Composition of Membrane Lipids

Growth conditions for the production of phospholipid reveals 10 bands in ^32^P- and ^14^C-autoradiography of two-dimensional TLC, under certain conditions, an eleventh band may be visible [12,18]. Variation of growth temperature (39 °C,49 °C,59 °C) shows (slight) differences in migration due to *– inter alia* - varying pentacyclization, consistent with [19,20,21,22]. Increasing the duration of growth until harvesting (48 h, 72 h, 96 h) did not significantly influence the composition and yield of membrane lipids. Variation of pH influenced lipid composition; MPL yield was highest at pH 2–2.5, lower at pH 1.5. Last but not least, the influence of varying amounts of yeast, glucose, and citrate added to the culture medium was determined. Highest yield in MPL is at 10 g/L glucose, 2 g/L yeast (twice reloaded during growth) and pH 2–2.5. For semi-continuous growth of several generations, in the beginning pH was set to 1.5 for “selective” culture conditions and re-adjusted up to the final generation (mostly 5 to 7 serial generations were applied). In similar way, culture conditions were also optimized for glycolipids. 

Conditions for optimal production of cell mass and the main tetraether phospholipid (MPL) which is correctly a gulopyranosyl-(*β*1-1)-caldarchaetidylglycerol [23], yields up to 80% of total phospholipid [12]. All other fractions amount only to 0.5–10%. 

Substituting glucose by citrate in the cultures diminished the amount of MPL by roughly 10% to about 65% [12]. Using Freundt’s solution instead of Darland’s medium [1] doubled the yield in cell mass. The complete composition of *T.a*. membrane lipids refers to [18]. 

Purification [24] yields highly purified MPL of 99% purity for further analysis and the production of model membranes. Longstanding discussions about pentacyclization of tetraether lipids started from the first report [25] with 62% zero, 37% two, and 1% four pentacycles at 40 °C growth temperature and 26% zero, 50% two, and 24% four pentacycles at 60 °C growth temperature per C_40_ biphytanyl chain. More differentiated pentacyclization for the whole caldarchaeol molecule consisting of 2 biphytanyl chains was presented by [21,22]. Different growth conditions and membrane fractions, variation in extraction and purification methods influence the number of pentacycles [19]; however, almost all reports have in common that increasing growth temperature increases the number of pentacycles [19,20,21,22]. Moreover, increasing pH was also reported to increase the number of pentacycles [21] (Table 1).

## 5. Differential Thermal Analysis (DTA) and Differential Scanning Calorimetry (DSC)

All DTA/DSC determinations [22,26,27,28,29,30,31,32,33,34] were conducted with hydrated lipids at various degrees of hydration, directly mixed into the analysis pans (details refer to the respective references), without checking planar or spherical membrane formation. Thus, all values presented are related to “hydrated lipid”.

All membrane lipid fractions of *T.a.* have their main phase transition below 0 °C. Enthalpy changes ΔH are about a magnitude smaller with TEL than with phase transitions of common bilayer-forming ester phospholipids (e.g., dipalmitoyl phosphatidylglycerol, DPPG) [26] and smaller for total lipid extract from *T.a.* than for fractions of phospho- and glycolipids (Table 2). When tetraether lipid (TEL) from the apolar fraction is admixed to MPL, ΔH decreases. Hence it is concluded that low ΔH value of the total lipid is also due to its content of apolar lipids [26,27]. 

The phase transition of the glycolipid fraction appears more complex than that of the other fractions. A metastable transition from −65 to −30 °C precedes the main transition in heating scans, the former immediately turning into the latter. Positive and negative ΔH (endotherm and exotherm) of this pretransition amount to zero. In total lipid of *T.a.* this metastable transition does not occur, although glycolipid is present; obviously it is suppressed by other components or fractions [26,27]. 

Lyotropic and thermotropic phases and phase transitions of glycolipids were extensively reviewed in [35]. Investigating amphiphilic bilayer-forming glycolipids the authors suggested that hydration of the head groups and the penetration of water into the interface between hydrophilic and hydrophobic regions determine the formation of complex non-lamellar phases and transitions to a highly ordered lamellar liquid-crystalline phase, which is rather determined by the shape of the hydrocarbon chains. Presumably, hydration of the headgroups and the hydrophilic-hydrophobic interface also influences the complex formation of metastable phase(s) and phase transitions observed in tetraether glycolipids, such as MGL.

Changes of pH (from 2 to 6.8) in the hydration buffer and various concentrations of sodium chloride, magnesium chloride or calcium chloride only slightly influence the main phase transition of MPL, not comparable to their strong influence on DPPG [26].

In the beginning of these experimental series, with MPL59 the glass transition from sub-A phase to A was detected and the transition behavior from A to C similar to MPL39 (Table 3). In the course of improved purification methods [24] and more detailed scanning procedures with sophisticated variations of heating and cooling scans, a more complex phase transition behavior was detected also with MPL59, especially metastable phase transitions [22]. Phase transitions were measured in detail of MPL extracted from cells grown at different temperatures (39 °C, 49 °C, 59 °C), i.e., with varying pentacyclization (as shown above in Table 1). MPL49 behaves like mixtures of MPL39 and MPL59 [28,29].

### 5.1. Cryoprotectants 

The influence of different cryoprotectants at varying concentrations and mixtures was determined (e.g., ethylene glycol, glycerol, DMSO, methanol, cacodylate) and correlations established concerning the shift of phase transitions and the change of enthalpy (ΔH) [30]. Cryoprotectants are necessary in DTA/DSC with tetraether lipids, because the main phase transition is below zero. The influence on the main phase transition was in a range of 20 degrees (between −20 °C and 0 °C); shape of the transition curve and ΔH were not influenced considerably. Above 0 °C scans were done also without cryoprotectants to make sure that there was no quenching effect [30]. In general, 0.38 M sodium cacodylate/HCl, pH 7.0 was used in DTA. 

### 5.2. Mixture of MPL with Other Lipids

MPL was mixed with other lipids (Table 4), diether lipids, methyl-branched diphytanyl-glucosylglycerol (DPhGG) and unbranched dipalmitylglucosylglycerol (DPGG) [27] and with ester lipids, DPPG and dipalmitoyl phosphatidylcholine (DPPC) [31].

Diphytanylglucosylglycerol (DPhGG) mixes with MPL at all molar ratios without forming domains or tendency to phase separation. The other three lipids differ in their behavior; they form also mixed phases with MPL, however with tendency to phase separation forming domains with high content and those with low content of the respective lipid. This does neither depend on ether or ester bonds of the lipid, nor on charges of the polar head group and is only marginally influenced by concentration of calcium ions or protons (pH) [27,31]. 

In comparison of the two ether lipids, MPL obviously exerts a condition between the branched diether lipid without phase transition (except for the rigid-glass transition) and sharp and intensive phase transition of the unbranched diether lipid at 62–65 °C [27]. The ester lipids investigated form mixed phases with MPL, but all of them tend to phase separation and to form distinct domains with high and low molar ratios [31].

### 5.3. Mixture with Cholesterol

Cholesterol is not a natural constituent of the cell membrane of *T.a*. Hence, its influence on the thermotropic behavior of MPL was investigated. In phospho-ester lipid bilayers cholesterol forms hydrogen bridges from its C3-hydroxyl to the ester carboxyl oxygen [36,37] reaching with its own molecular length to the middle of the bilayer with different effect on the latter above or below phase transition: in the solid-analogue gel phase cholesterol decreases lipid cooperativity and membrane order, whereas in the liquid-crystalline phase it increases the order degree [36,37]. 

In ether lipid membranes cholesterol cannot form this kind of hydrogen bonds. In case of MPL it is suggested that cholesterol C3 hydroxyl forms hydrogen bridges to the phosphoester oxygen. This means that cholesterol does not insert into the depth of the membrane as far as with ester lipids [35].

It was interesting to see whether the influence of cholesterol differs between MPL39 and MPL59. For DTA preparation, MPL and cholesterol were dissolved separately in chloroform/methanol and mixed into the pans with hydration buffer under nitrogen. MPL showed the well-known weak phase transition behavior between −30 and −5 °C with T_m_ at −10 to −15 °C. Cholesterol diminished the height of phase transition endotherm and shifted T_m_ to −23 °C. 

MPL39 shows more complex behavior than MPL59; in general, cholesterol shifts phase transitions to lower temperatures and reduces heat flows. Along with increasing cholesterol fractions the steepness of the exotherms is reduced indicating increasing time constants of the transition from A/C to B. The endothermal transition of from B to C, at +20 °C is especially influenced by cholesterol. A fraction of cholesterol remains in the stable gel phase of MPL; at high cholesterol fractions (e.g., c_chol_/c_MPL39_ = 1.8) additional endothermal-exothermal transitions occur from −8 to +3 °C and from +3 to +10 °C, which indicates a second mixed phase between cholesterol and MPL, a domain with higher cholesterol fraction than in phase B. Modification of the heating scans confirms the kinetic effect of cholesterol on the time constant of the transitions of MPL39. Enthalpy changes are also modified by cholesterol depending on the molar ratio; ΔH of the transition from A to C is linearly reduced with increasing fractions of cholesterol. Extrapolation to ΔH = 0 results in c_chol_/c_MPL59_ = 1.9 and c_chol_/c_MPL39_ = 3.0. Extrapolation to ΔH = 0 of the MPL39 transition from B to C confirms the molar ratio of 3.0. 

Taking into account the roughly double molar mass of membrane spanning MPL compared to common bilayer-forming lipids, the value found for MPL59 corresponds to 1,2-distearoyl-*sn*-glycero-3-phosphocholine (DSPC) and 1.2-diloeoyl-*sn*-glycero-3-phosphatidylcholine (DOPC) [38], to a saturated C16 cerebroside [39], and is slightly higher than the value found for 1.2-dipalmitoyl-*sn*-glycero-3-phosphatidylcholine (DPPC) [37]. 

The value of MPL39 (3.0) is clearly higher than of MPL59 (1.9), which is interpreted in a way that less pentacyclization enables the incorporation of higher amounts of cholesterol. Cholesterol forms mixed phases with MPL in its liquid-crystalline and its metastable gel-analogue gel states, the latter being stabilized by cholesterol. At high cholesterol fractions (c_chol_/c_MPL39_ = 1.8) new metastable phases occur with domains of varying cholesterol contents [32].

### 5.4. Mixture with Pore-forming Peptides and Ionophores 

Various synthetic α-helical peptides with increasing chain lengths (P10, P11, P17, P20) were investigated [33], as well as alamethicin, melittin, valinomycin and nonactin [34]. Each of these compounds was incorporated into the hydrated lipid structure of MPL. Depending on the concentration (c_pep_/c_MPL_), the influence on ΔH increased with increasing chain lengths. On the other hand, P17 modified T_m_ most, clearly more than all other peptides investigated, including P20. 

All four peptides have a lipophilic C-terminus in common, (Ala-Aib-Ala-Aib-Ala)_2_-*O*-methyl, and an N-terminal positive charge: P10, H-(Ala-Aib-Ala-Aib-Ala)_2_-O-Me HCl; P11, H-Gln-(Ala-Aib-Ala-Aib-Ala)_2_-O-Me; P17, H-Ala-Leu-Ile-Leu-Leu-Ala-Gln-(Ala-Aib-Ala-Aib-Ala)_2_-O-Me; P20, H-Asn-Arg-Arg-Ala-Leu-Ile-Leu-Leu-Ala-Gln-(Ala-Aib-Ala-Aib-Ala)_2_-O-Me HCl (all compounds were synthetized by Dr. G. Becker, University Tübingen).

Among the pore-forming compounds studied, melittin influences ΔH and T_m_ considerably stronger than the other ones, alamethicin and valinomycin are similar, nonactin appears weaker. 

Concerning T_m_, valinomycin differs from the other compounds, whereas nonactin < alamethicin << melittin shift T_m_ to lower temperatures (concentration dependent), valinomycin shifts T_m_ to higher temperatures; however, the effect does not increase with increasing molar fractions. Extrapolation to ΔH = 0 indicates how many lipid molecules are disturbed by one molecule of ionophore (Table 5).

The following classification exists according to the effects on T_m_ and ΔH [40]:

Group 1 increases ΔH. These compounds interact mainly electrostatically with polar head groups and do not penetrate the hydrophobic region of the membrane. General examples are polylysine, ribonuclease and from the compounds investigated, nonactin fits partially into this group, but exerts also some features of groups 2 and 3.

Group 2 strongly decreases ΔH and T_m_. These compounds are bound to polar head groups via ionic interaction and subsequently partially incorporated into the lipid layer of the membrane disturbing lipid cooperativity and membrane structure. A general example is cytochrome C and among the compounds investigated, melittin and peptide 17.

Group 3 shows concentration-dependent decrease of ΔH and varying influence on T_m_. These compounds are integrated into the hydrophobic region of the membrane and may be membrane spanning. General examples are gramicidin A and glycophorin, among the compounds investigated, alamethicin, valinomycin and the peptides P10, P11, and P20.

## 6. Model Membranes

### 6.1. Black Lipid Membranes

Black lipid membranes were first described by [41], the technique has often been criticized, modified and improved, which is not the topic of this review. However, where necessary, readers will be referred to the respective literature. 

After it was clear from DSC/DTA that the abovementioned peptides and ionophores mix with MPL, conductance mediated by valinomycin, nonactin, and gramicidin were investigated in self-assembling black lipid membranes [42]. Before performing these experiments, conditions for producing stable BLM with MPL had to be established. To this end, BLM assembly of various lipids was compared: total polar lipid fraction (TPLF) from T.a. (TPLF-Ta), MPL, MGL, GL hydrolyzed from MPL, LPS, with glycerol-dialkyl-nonitol-tetraether from *S. acidocaldarius* (GDNT, kindly provided by Dr. A. Gliozzi, Camogli, Italy), DPhPC, and DPhGG (kindly provided by Dr. L. Six, Regensburg, Germany). Details about the formation and the stability of the BLM from these lipids were described in [42].

TPLF-Ta, MPL, MGL, GDNT and DPhPC are able to form BLM which are stable for 5–6 h; glycolipid obtained by hydrolysis of MPL and DPhGG form BLM stable up to one hour, and lipopolysaccharides (LPS) from *T.a.* do not form BLM. It was known from [43] that tetraether lipids (GDNT) can form BLM, demonstrated by two photos of successive moments of BLM formation with GDNT from squalene dispersion at 72 °C. From their experiments with squalene or more complex solvent mixtures the authors concluded that GDNT can form BLM only above 40 °C [43]. Experiments by [42] were conducted at RT (22 °C) producing stable BLM with GDNT from 1% dispersion in *n*-decane and torus formation with 2 µL of 0.25% DPhPC in the same solvent [42]. 

The first criterion of the membrane properties is the solvent from which BLM are produced; *n*-hexane, *n*-decane, and squalene were used. The second criterion is the annulus or torus formation, which is the connection of the BLM to the rim of the pore in the Teflon separation plate [44]. The torus can be formed from the same or from different lipid. The formation of BLM and annulus with bilayer-forming lipid under various conditions was well demonstrated in schematic drawings [45,46]. 

In general, the torus is formed by a small amount of bilayer-forming DPhPC (2µL of a 0.25% solution in *n*-decane) laid around the rim of the aperture. A 1.0% solution of TPLF-Ta in *n*-decane for the BLM porus yields most stable results (5–6 h). However, BLM stable for up to one hour, can be formed from MPL or MGL (0.75% solution in *n*-decane), if the torus is formed from the same small amount of TPLF-Ta from 0.25–0.5% *n*-decane solution. This observation indicates that tetraether lipid is able to form a torus by laying around the Teflon rim in a very flexible manner, presumably including “horse-shoe” formation. In analogy to [45,46], it can be assumed that tetraether lipid molecules are not standing upright in the torus, different from the straight formation in the porus. 

Capacitance and dielectric thickness of BLM were determined: for tetraether lipids, these parameters do not depend on the organic solvent used, different from bilayer-forming lipids DPhPC and DPhGG; thickness increases from squalene via *n*-decane to *n*-hexane. The reason is obviously that *n*-hexane widely remains in the hydrophobic center of the bilayer leading to “swollen” BLM, *n*-decane also remains in the bilayer, however at lesser extent, and squalene is pressed out of the membrane, because of its molecular structure and size [47,48]. 

From these results it can be assumed that in BLM, MPL and MGL are oriented in upright membrane-spanning position with a stable dielectric thickness of about 2.5 nm, no matter which solvent is used. The *in-silico* model of four tetraether lipid molecules results in calculated membrane thickness of the hydrophobic region between the two glycerol residues to 2.1–2.3 nm [17]. To evaluate differences in pentacyclization in this model, the influence of the number of pentacycles was calculated: none 22.95-22-97 Å, three pentacycles 22.48-22.56 Å, and five pentacycles 21.17-21.18 Å [17].

The slight differences result from two variations of pH; pH 1.5 as the preferred growth temperature used in fermenter cultivation of *T.a*. (close to the growth optimum at pH 2) and pH 4, which was demonstrated as the highest membrane stability in lysis experiments [16,17]. The lower values (21.17; 22.48; and 22.95 Å) are calculated at pH 4, the higher ones (21.18; 22.56; and 22.97 Å) at pH 1.5. At the point of highest stability (pH 4), the cell membrane of *T.a*. appears a little bit compacter in this computer simulation than at growth condition of pH 1.5. This seems to be in line with [20]. 

In general, stability of BLM decreases with increasing conductance. Although LPS from *T.a*. do not form BLM by themselves, they drastically decrease conductance mediated by ionophores [42] (Table 6).

### 6.2. Monomolecular Films at Water/Air Interface

**Figure 4:**
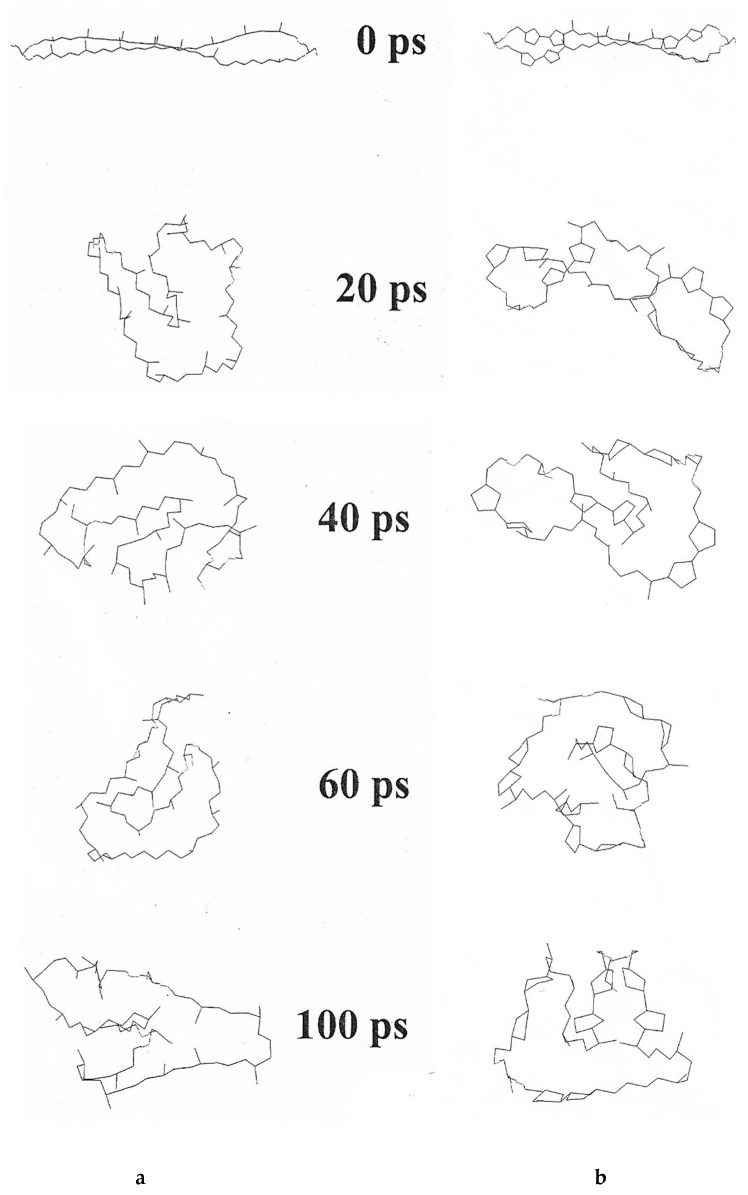
Computer simulation of the dynamics of a single MPL molecule in vacuo. Computer simulation of the dynamics of a single MPL molecule in vacuo without any further restriction was set at 37 °C and a time course of 100 psec (ps). (**a**) MPL without pentacycles; (**b**) molecule with a total of eight pentacycles [22].

Monomolecular films at the water/air interface were conducted with and compared from three ether lipids, MPL, DPhG, DPhGG [49]. Collapse pressure of MPL was 39 mN/m^2^, DPhG 25 mN/m^2^, DPhGG 48 mN/m^2^, values correspond to those published for ester [50] and ether lipids [51], in case that collapse occurred directly from the liquid-expanded state of the lipids. From liquid-condensed state, collapse pressures for these lipids were around 70 mN/m^2^. The calculated molecular area at this collapse pressure is 0.73 nm^2^ for all three lipids investigated. If so, it was concluded that MPL is in an upright position under these experimental conditions. Elferink et al. [52] had reported 0.82 nm^2^ for GDNT from *S. acidocaldarius*, which indicates upright orientation of the membrane spanning tetraether lipids, too. On the other hand, Yamauchi et al. [53] had reported a molecular area of 0.9–1.4 nm^2^ for synthetic tetraethers with one membrane-spanning chain and two shorter single chains. Horse-shoe orientation was considered at least for part of these molecules and was first theoretically postulated for MPL, too, especially from the computer model of a single MPL molecule with and without pentacycles [22]. This *in-silico* molecule model in vacuo, which – non-hydrated and without any other restrictions – immediately coils within 20–40 psec, the molecule without pentacycles only marginally faster than the molecule with maximal pentacyclization of eight pentacycles. Within 100 psec some minor reorientation of the coiling state occurred, always with both hydrophilic ends in close vicinity. The differences are much smaller than expected (Figure 4). The computer model of MPL is based on the chemical formula of gulopyranosyl-(*β*1-1)-caldarchaetidylglycerol [22,23,24]. 

With a Langmuir trough connected to a fluorescence microscope, the complex orientation of MPL was demonstrated with two distinct heights, one of 4–5 nm representing domains with upright position and another one of 1.5–1.8 nm forming “horseshoes” at the water-air interface and measured by atomic force microscopy (AFM) after Blodgett-transfer to solid surface [54]. The membrane height of 4–5 nm correlates to the dielectric BLM thickness of 2.5–3.0 nm, which is the hydrophobic non-conductive moiety without the polar head groups. In the above-mentioned computer-simulated membrane model [17], this was calculated as the distance of ± 2.2 nm between the two glycerol residues at either side of the caldarchaeol.

### 6.3. MPL Liposomes

**Figure 5:**
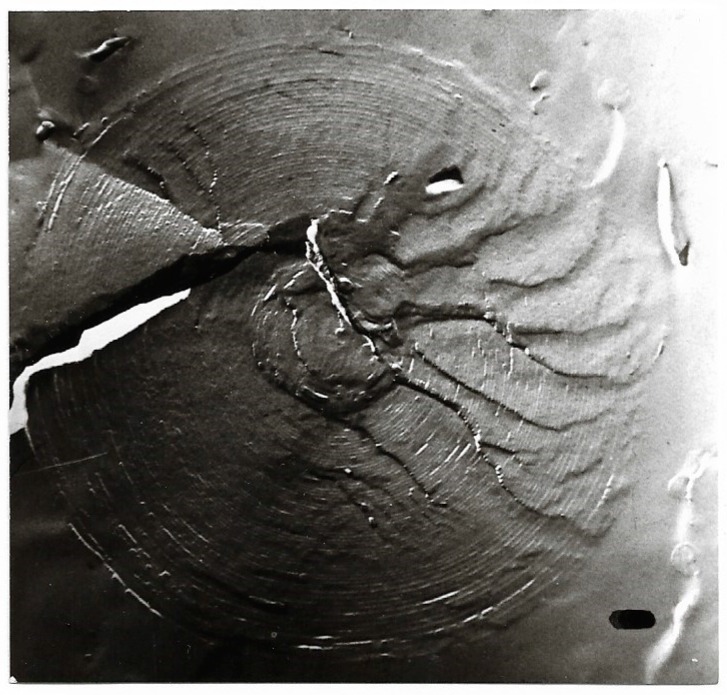
Freeze-fracture electron microscopy of a giant multilamellar, densely packed structure under poorly hydrated lyotropic condition; magnification ×54,000; bar = 100 nm. The formation resembles - though spherical - the P phase of GDNT in [58,59,60]. Electron micrographs of Figure 5, Figure 6, Figure 7, Figure 8, Figure 9 and Figure 10 had been presented in [13,56,61].

MPL forms stable unilamellar and multilamellar liposomes in the range of 100–600 nm depending on the preparation method; hand-shaken multilamellar MPL liposomes without further processing are larger, in µ-meter range (Figure 5). Zeta potential of −58.2 mV was determined from liposomes of 80 and 150 nm in diameter, at 137.4 V in 75 mM NaCl, 5 mM sodium phosphate, pH 7.4 (corresponding to a 1:1 dilution with distilled water of the original liposomal suspension in phosphate-buffered saline) [55]. From these values, one elemental phospho-acid negative charge per 14 to 15 nm^2^ on the liposomal surface is calculated. In other words, the majority of MPL molecules are oriented with their sugar residues facing outwards, approximately at a ratio of one charge per 15 to 20 MPL molecules with their sugar molecules at the liposomal surface.

All classical preparation methods known for bilayer liposomes have been successfully applied also for MPL. Liposomes are stable at RT for more than six months and, stored at 4 °C, even for 2 years [13,55,56,57].

**Figure 6:**
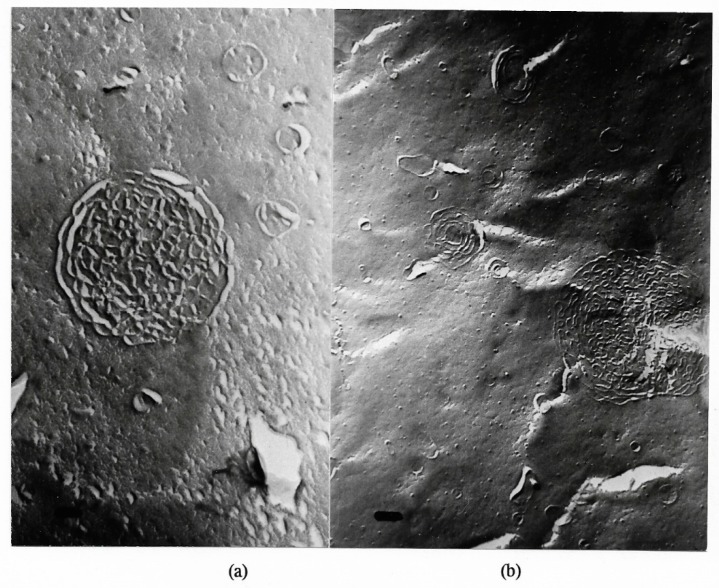
Freeze-fracture electron microscopy of a multilamellar hand-shaken liposome (**a**) after two and (**b**) after 4 passages through the French pressure cell, in the process of disruption into smaller units; magnification ×67,500; bar = 100 nm.

**Figure 7:**
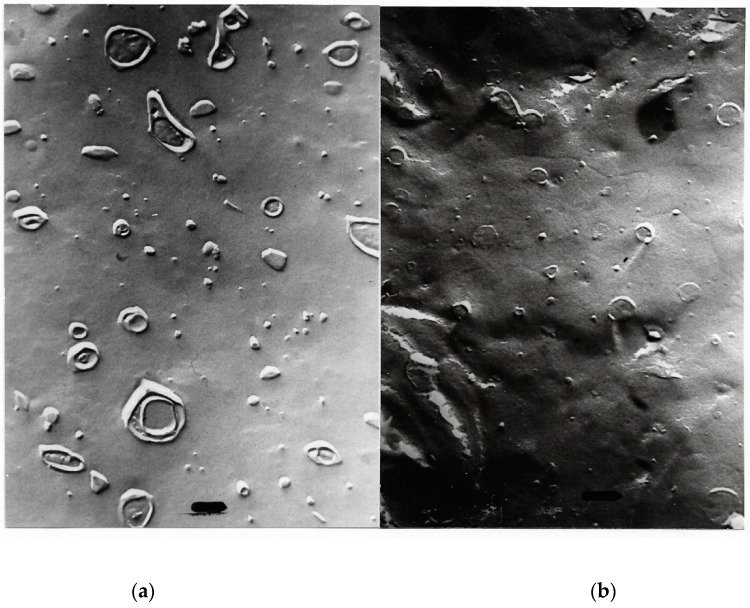
Freeze-fracture electron microscopy of mainly unilamellar liposomes after 5 (**a**) and 6 (**b**) passages through the French pressure cell; magnification ×67,500; bar = 100 nm.

Transitions of large multilamellar to smaller unilamellar vesicles are depicted in Figure 6 and Figure 7: 

Tetraether lipid liposomes from MPL had been characterized in [13,55,56,57] (Table 7, Table 8 and Table 9). For reconstitutions and other biochemical experiments, liposomes between 100 and 500 nm in diameter were used (Table 7).

Order parameters of liposomal membranes were determined by electron paramagnetic resonance (EPR) with spin labels successively reporting from the outer polar-to-hydrophobic interface of the membrane to the inner hydrophobic moiety (Table 8). 

Comparison with egg lecithin liposomes demonstrates that the outer region of the MPL membrane is considerably more rigid than in the lecithin membrane. Differences become smaller, the deeper the reporter group inserts into the hydrophobic membrane moiety. Hydrophobic 5-doxyldecane exerts still higher order in MPL than in lecithin, but the smaller hydrophobic di-*tert*.-butylnitroxide shows slightly lower order or higher fluidity in MPL than in lecithin membranes. In other words, the fluidity gradient across the liposomal MPL membrane is higher than that across the liposomal lecithin membrane [13,55].

Bile acid salts were mixed at roughly physiological molar ratio, chenodeoxycholic acid: cholic acid: deoxycholic acid, 2:2:1, and applied at about physiological concentration of 10 mM and at very high, 30 mM, concentration. Different incubation times between 5 and 60 min did not essentially influence the results [68] (Table 9).

### 6.4. Mixed Liposomes

Thermotropic DTA/DSC experiments had shown that MPL mixes with bilayer-forming lipids at certain molar ratios. Hence, it was examined whether mixtures of MPL and egg lecithin are able to form stable liposomes. These liposomes were prepared with *n*-octylglucoside detergent dialysis, evaluation was accomplished with size, shelf (size) stability and freeze-fracture EM. The range given in Table 10 denotes results with different MPL preparations; various purification steps were tested, via one column and via two subsequent columns ending up in the purification published by [24]. 

Shelf stability was tested at 4 °C for 2 years: MPL liposomes were stable for 2 years, egg lecithin liposomes only 2–3 months. Mixed liposomes of 25% MPL and 75% egg lecithin were stable for clearly more than 6 months, determined in the laser particle sizer. 50/50% and 75% MPL/25% egg lecithin did not yield stable liposomes. There was no homogenous distribution detected in the laser particle sizer, but several distinct peaks indicating separation into MPL and egg lecithin [13,55,69]. EM confirmed differently breaking liposomal membranes; furthermore, multilamellar structures, obviously both spherical and planar, appeared. At mixture 50/50%, lecithin liposomes with MPL domains were visible. Conclusion from these experiments: liposomal mixtures with lecithin should not exceed 25% MPL.

**Figure 8:**
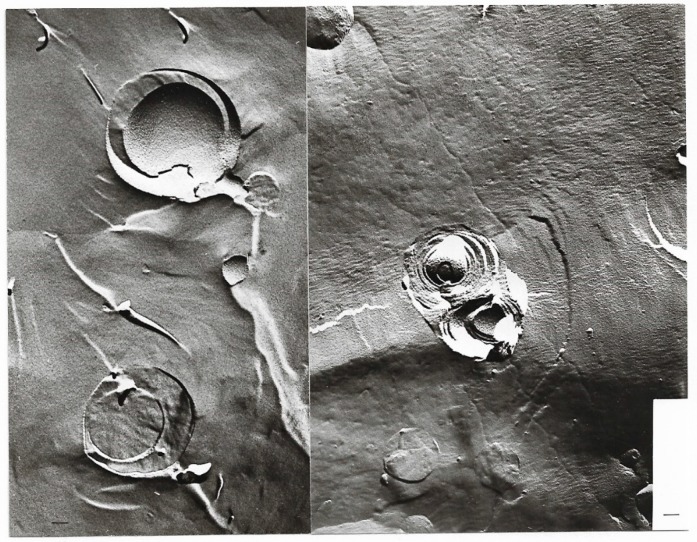
Freeze-fracture electron microscopy of mixed liposomes consisting of 25% MPL and 75% egg lecithin after 89 days; magnification ×67,500; bar = 100 nm.

**Figure 9:**
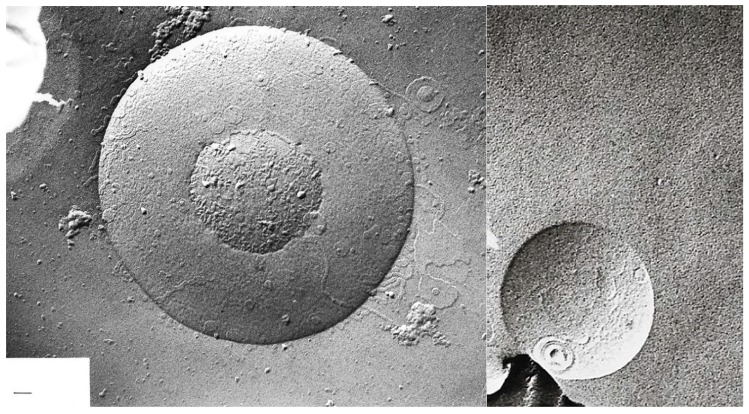
Freeze-fracture electron microscopy of mixed liposomes consisting of 50% MPL and 50% egg lecithin; MPL domains can be seen in the egg lecithin liposomes; magnification ×67,500; bar = 100 nm.

**Figure 10:**
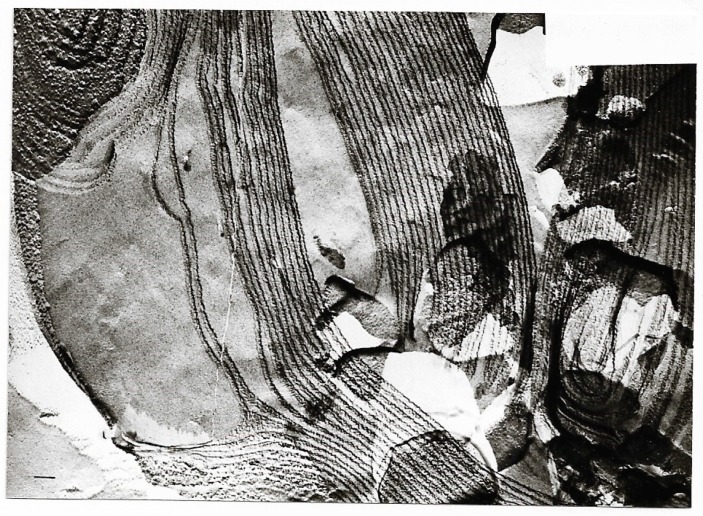
Freeze-fracture electron microscopy of mixed liposomes consisting of 75% MPL and 25% egg lecithin; planar structures develop because of the instability of mixed liposomes; magnification ×67,500; bar = 100 nm.

Representative electron micrographs [56,61] from Table 11 are depicted in Figure 8, Figure 9, Figure 10.

Swelling rates (OD λ = 570 nm) were determined with addition of glycerol (C-3), erythritol (C-4), xylitol (C-5) and mannitol (C-6). As expected, swelling occurs in the series glycerol > erythritol > xylitol > mannitol. MPL liposomes exert lowest swelling, in all cases under 3%. In mixed liposomes with increasing amounts of bilayer-forming phospholipids (DPPC or phosphatidylinositol up to 20%) swelling under the same conditions increases to almost 70% [70]. Carboxyfluorescein (CF) release was measured as percentage of increase in fluorescence (Table 12).

Furthermore, the amounts of alcohols (methanol, ethanol, *n*-propanol) for 100% CF release were compared; liposomes of egg lecithin and DPPC did not differ essentially, whereas with MPL the percentage of the alcohols (% *v/v*) had to be roughly doubled. With sodium deoxycholate (DOC) molar concentration had to be doubled, too. Sodium dodecyl sulfate (SDS) did not manage to release more than 10% CF from MPL liposomes at any tested concentrations up to 5 mM; 0.5 mM concentration was enough for more than 50% release from DPPC liposomes, similar to egg lecithin with 0.45 mM; Triton-X100 needed 0.0023% with egg lecithin liposomes, 0.0075% in DPPC liposomes, and 0.02% with MPL liposomes. 

Although CF leakage was much lower from MPL liposomes than from those made of egg lecithin or DPPC [70], hydrophilic CF was transferred very well from MPL liposomes to trypsinated T84 tumor cells and rapid intermembrane exchange of lipophilic bromobimane and Nile red from MPL liposomal membrane into isolated red cell membrane (ghosts) was demonstrated [71].

### 6.5. Proton Permeability and Light-Driven Proton Pumping

#### 6.5.1. Proton Permeability

Proton permeability of liposomal membrane was measured via potassium diffusion in the presence of valinomycin in a system only containing H^+^ and K^+^ as permeable ions. Internal K^+^ was determined by potassium-binding (benzofuran) fluorescence indicator (PBFI). An electric potential of 186 and 101 mV was applied and extrapolated to zero. 

Potassium uptake into the liposomes through valinomycin is only possible if proton efflux counteracts the increase of positive charges in the liposomal lumen. Hence, proton permeability coefficients can be calculated from increasing PBFI-K^+^ fluorescence. 

Potassium diffusion through liposomal MPL membrane was only measurable in the presence of excess valinomycin. Since only protons could counteract potassium diffusion, proton permeability coefficients could be calculated, for MPL liposomes 4.5 × 10^−6^ cm s^−1^ versus 10^−5^ to 10^−4^ cm s^−1^ in egg lecithin liposomes. Further addition of valinomycin only increased diffusion marginally in MPL liposomes and additional palmitic acid, which stimulates diffusion in egg lecithin liposomes, did not considerably influence ion permeability of MPL liposomes. Only the combination of valinomycin with the potent uncoupler FCCP resulted in enhanced potassium diffusion via valinomycin and proton counter flux via FCCP.

Proton permeability was 2.7 × 10^−5^ nmol H^+^ s^−1^ cm^−2^ in MPL liposomes versus 1.2 × 10^−4^ s^−1^ cm^−2^ in egg lecithin liposomes, i.e. lower in MPL liposomes by a factor of ~50 (Table 13). Rate constants (Ksec^−1^) rapidly further increased in egg lecithin liposomes with temperature, but not in MPL liposomes [72].

Proton permeability had been reported on liposomes of TEL from *Sulfolobus acidocaldarius* [73]. Although exact comparison of different methods is difficult and values of rate constants not calculated in [73], they can roughly be estimated from the comparable figures in [72] and [73] at 70 °C; they appear clearly smaller in MPL liposomes than in liposomes from TEL extracted from *Sulfolobus acidocaldarius.* The reasons for this difference have not been investigated, but in MPL liposomes stability increases with higher purity of TEL [13,69]. Liposomes for measurement of proton permeability and reconstitution experiments were made from 99% pure MPL according to [24]. On the other hand, extremely low proton permeability coefficients of liposomes from the polar lipid fraction E (PLFE) of *Sulfolobus acidocaldarius* were reported, (0.3–0.5) × 10^−8^ cm s^−1^ [74], which is even one magnitude lower than in MPL liposomes.

#### 6.5.2. Reconstitution of Bacteriorhodopsin (BR) and Light-Driven Proton Pumping

Bacteriorhodopsin was reconstituted into liposomes from MPL to measure light-driven proton flux in the absence and presence of ionophores and uncouplers. Subsequently, bacteriorhodopsin was co-reconstituted together with ATP synthase from *Micrococcus luteus* to measure light-driven ATP synthesis in an artificial liposomal system.

Molar ratios between protein and lipid was between 1:15 and 1:25 for the reconstitution of monomeric bacteriorhodopsin [75], which is in the range reported for amphiphilic lipids [76]. Various reconstitution methods were applied, best results were obtained by the detergent method [65], subsequent removal of the respective detergent by dialysis and saccharose gradient centrifugation [72,75]. For the reconstitution into tetraether lipid, essentially higher detergent concentrations were necessary than for amphiphilic bilayer-forming lipid, in case of *n*-octylglucoside, 60 mM instead of 40 mM, in case of TODC the concentration had to be doubled.

Monomeric bacteriorhodopsin (BR) was reconstituted into MPL liposomes. Illumination of BR-proteo-liposomes was carried out in a volume of 1.3 mL containing 354 µg MPL and 30 µg monomeric BR and led to an initial increase of pH in the outer medium of 0.06 units (Δ pH). When the light was switched off, a minute (almost not measurable) decrease of pH occurred, quickly reaching a plateau. At least 0.02% triton X-100 were necessary to reach the original pH value. The experiment shows that there is some light-driven pump activity of BR into the liposomes and almost no proton efflux through the membrane and/or through BR.

The initial pump rate was increased 30-fold [ng H^+^ (mg BR × min) ^−1^] by addition of valinomycin (0.24 µM, molar ratio valinomycin/MPL = 1:692), under these conditions proton efflux was 0.15 ng H^+^ (mg MPL × min) ^−1^. Further increase of valinomycin concentration did not change this result essentially. Addition of gramicidin (0.4 µM) decreased both, the initial pump rate and proton efflux, almost by factor 10 (Table 14).

#### 6.5.3. Light-Driven Liposomal ATP Synthesis

Co-reconstitution was accomplished by a ratio 1:1:20 (mol monomeric bacteriorhodopsin: mol ATP synthase from *Micrococcus luteus*; mol lipid), detergents *n*-octylglucoside or TDOC, tauro-deoxycholate. Although co-reconstitution into MPL liposomes did not yield highest synthesis rates among the lipid systems tested (Table 15), the advantage of MPL was its superior liposomal stability as compared to the other lipids.

## 7. Applications

A wide range of biomedical and biotechnological applications of TEL and liposomes of TEL has been presented earlier [7,55,71] including oral delivery of vaccines and acid-sensitive pharmaceuticals [56,81]. Chemical modification resulting in cationic tetraether lipids for transfections was reported [82]. Based on the co-reconstitution experiments [72,75,76,77,78,79,80,83,84], protein synthesis by an artificial ATP-producing photosynthetic cell was recently reported [85] using soy phosphatidylcholine for the liposomal membrane. Although the latter was claimed sufficiently stable for experiments, for technical application, further stabilization of the reconstitution membrane with TEL/MPL may be necessary or at least advantageous.

## Figures and Tables

**Table 1:** Average number of pentacycles in the main glycophospholipid (MPL).

pH	1.2	1.5	1.8	2.4	3.0
**Growth Temperature**					
**39 °C**		2.8 [22,24]			
**40 °C**			3.2* [20]		
**45 °C**			3.6 [21]		
**50 °C**			3.8* [20]; 3.9 [21]		
**55 °C**	4.0 [21]		2.8 [18]; 4.1 [21]	4.8 [21]	5.1 [21]
**59 °C**		3.8 [22,24]			
**60 °C**			4.2* [20]; 4.5 [21]		

**Footnote:** * Average pentacyclization is given in [20] for one biphytanyl C_40_ chain and the value for one caldarchaeol molecule is obtained by multiplication by a factor of 2. Specific calculation methods for average pentacyclization are given in the respective references.

**Table 2:** Phase transitions of lipid fractions from *Thermoplasma acidophilum.*

Growth at 59 °C; Lipid Fraction From *T.a.*	Glass Transition Range [°C]	Metastable Phase Transition Range [°C]	Main Phase Transition Range [°C]	T_m_ [°C]	ΔH [J/g]
Total lipid	−95/−50		−40/−10	−16.5	5.0
Phospholipid	−95/−50		−34/−8	−14.3	8.9
MPL	−95/−50		−32/−7	−14.0	8.9
Glycolipid	−95/−65	−65/−30	−30/−13	−17.7	9.0

**Footnote:***T.a., Thermoplasma acidophilum,* values adapted from [26].

**Table 3:** Phase transitions of MPL at different growth temperatures.

MPL	39 °C			49 °C			59 °C		
Scan	dT/dt [K/s] phase	°C	endo-/exo-therm	dT/dt [K/s); phase	°C	endo-/exo-therm	dT/dt [K/s]; phase	°C	endo-/exo- therm
heating	0.02–0.13	−30 → +30	endo-exo-endo	0.017–0.083	−30 → +20	endo-exo-endo			
T_m_	A→C	−15	endo	A→C	−15	endo	A→C	−15	endo
	A/C→B’	0	exo	A/C→B’	~ 0	small exo			
	B’→C	20	endo	B’→C	20	small endo		+17	endo
ΔH	depends on dT/dt			depends on dT/dt			widely indep-endent		
cooling	0.08 C→A	−20 → −45	exo	0.033 C→A	−30 → −50	exo	0.033 C→A		exo
T_m_								−27	exo

**Footnote:** Phases and phase transitions: C = liquid crystalline phase, stable above 20 °C; A = metastable gel phase, obtained by fast cooling of C (exothermal transition between −20 and −45 °C), occurring in heating scans between −30 and −5 °C; B = stable gel phase (the formation of B is estimated to take minutes, which cannot be accomplished in DTA/DSC scans, hence incomplete phases of B are formed, here denoted B’. Possibly, a second incomplete phase B exists depending on variation of the scans. For further details and phase transition models, reference is made to the original literature [22,26,28,29].

**Table 4:** Mixture of MPL with other lipids.

	Phase Transition				From	to	Mixture
		T_m_ [°C] range	J/mol	kJ/mol K			
MPL	Glass transition	−90/−50		1.26	Rigid glass	Gel analogue	
MPL	Main transition	−30	−14		Gel analogue	Liquid crystalline	
DPhGG	Glass transition	−84/−61		0.74	Rigid glass	Liquid crystalline	Unlimited, no phase separation
DPGG	sharp	62	−98				Limited; only >62 °C; <62 °C phase separation with DPGG-rich and DPGG-poor domains
DPPG	Transition and mixture behavior similar to DPGG						
DPPC	weak pretr, sharp ph.tr.	35/ 42					Limited, phase separation with DPPC-rich (ph.tr. >0 °C) and DPPC-poor (ph.tr. at −13 °C) domains

**Footnote:** pretr, pretransition; ph.tr., phase transition; values adapted from [27,31].

**Table 5:** Mixture of MPL with ionophores.

	Shift of T_m_ [°K]		MPL/Ionophore Ratio at ΔH = 0	Times 2^#^	DPPC/Ionophore Ratio at ΔH = 0
	MPL	DPPC/DHMG*			
**Alamethicin**	−4.7	−4.2	0.5	1.0	1.5
**Melittin**	−15.8	−10 to −20	2–2.5	4–5	6–8
**Valinomycin**	>0	−0.6	0.5	1.0	0
**Nonactin**	−3.4	−6.7/−2.7*	~0.3	~0.6	ΔH increases

**Footnote:** DPPC, dipalmitoyl phosphatidylcholine; * synthetic ether glycolipid 1,2-dihexadecyl-3-*O-β*-D-maltosyl-*sn*-glycerol (DHMG) was a gift from Dr. Six, University Regensburg, Germany. # for comparison with bilayer-forming lipid, the value of MPL is multiplied by a factor of 2; values adapted from [34].

**Table 6:** BLM and ionophores.

Lipid	Solvent	*C*_m_ [µF/cm^2^]	t_d_ [nm]	Stability	Conductance [µS/cm^2^]		
					Valinomycin 10^−7^ M	Nonactin 10^−6^ M	Gramicidin 5 × 10^−11^ M
MPL	*n*-decane or squalene	0.744	2.5 (3.0)	+	200–250	300	60
MGL			2.5 (3.0)	+/(+)*	700 (1000)*		110
GDNT		~0.7	2.5–3.0	+	1200	n.d.	130
DPhGG	*n*-decane	0.412	~4.5	(+)	125		
	squalene	~0.7	2.6–3.0	−			900
DPhPC	*n*-decane	0.412	4.72 5.68	+	185 85	50	40/double conc. 100
	squalene	~0.7	~3.0	(+)	480		1100

**Footnote:** *MGL isolated from *T.a*. membranes forms stable BLM (5–6 h) and valinomycin mediates conductance of 700 µS/cm^2^, whereas glycolipid obtained by hydrolyzing the phosphoester of MPL forms less stable BLM (up to one hour) and conductance mediated by valinomycin is up to 1000 µS/cm^2^. In general, potassium carriers, valinomycin and nonactin, exert higher conductance in tetraether lipid BLM than in bilayers, whereas pore-forming gramicidin reaches roughly 10-fold higher conductance in bilayers, provided dielectric thickness is comparable to that of tetraether lipid BLM. *C*_m_, membrane capacitance; t_d_, dielectric membrane thickness; values adapted from [42].

**Table 7:** Characteristics of MPL liposomes [55].

Preparation	Mean Diameter [nm] ± SD	Dispersity Index 0-1	Reference [62]
Hand-shaken	2500 to > 7500	0.9	[63]
Sonication	600 ± 40	0.5	[64]
Detergent dialysis	370 ± 35	0.5	[65]
French pressure cell	151 ± 22	0.4	[66]
Polycarbonate filter extrusion 200 nm	221 ± 63	0.6	[67]
Polycarbonate filter extrusion 100 nm	120 ± 40		

**Table 8:** Order parameters [13,55].

Spin Probe	Lecithin Liposomes	MPL Liposomes
3-Doxyl-5α-cholestane	0.655	0.932
5-Doxylstearic acid	0.644	0.781
16-Doxylstearic acid	0.201	0.258
5-Doxyldecane	0.130	0.187
di-*tert*-Butylnitroxide	0.117	0.091

**Table 9:** The effect of bile acid salts on liposomal size.

Liposomes	Egg Lecithin			MPL		
	Original	Bile Acid Salts		Original	Bile Acid Salts	
		10 mM	30 mM		10 mM	30 mM
size	159.8 nm	47.2 nm	1898.4 nm	131.5 nm	107.9 nm	114.8 nm

**Footnote:** Values adapted from [68].

**Table 10:** Mixed liposomes, average size.

Lipid/Detergent Ratio	MPL	MPL/EL 75:25 (% g/g)	MPL/EL 50:50 (% g/g)	MPL/EL 25:75 (% g/g)	Egg Lecithin
0.05	372 ± 98	558	−	490	−
0.1	446 (396–495)	396 (199–550)	728 (359–778)	484 (422–645)	−
0.15	n.d.	263 (169–307)	431 (386–664)	471 (453–489)	−
0.2	444 (421–466)	461 (480–792)	818	413 (325–510)	153 ± 5 (145–162)
0.3	−	−	−	−	144 ± 9 (137–150)

**Footnot:** Preparations which did not yield measurable results are not listed; EL, egg lecithin; average size values [nm] are adapted from [69].

**Table 11:** Shelf stability of mixed liposomes, average size.

Days	MPL		MPL/EL 75:25 (%g/g)	MPL/EL 50:50 (%g/g)	MPL/EL 25:75 (%g/g)	Egg Lecithin
0–2	466	372	408	386		159
5–10	450			394	396	146
15–38		358	695 (day 38)	395	411	149
49–70	446		−	366 (day 49)	405	149 (day 66)
80–90			−	−	446 (day 89)	−
128		434	−	−	-	−
746	498		−	−	-	−

**Footnot:** Values of average liposomal size [nm] are adapted from [69].

**Table 12:** Carboxyfluorescein release (% increase of fluorescence).

Lipid	4 °C	30 °C	50 °C
Egg lecithin	9%	41%	100%
DPPC	6%	32%	100%
DPPC/MPL molar ratio 2:1	5%	8%	62%
DPPC/MPL molar ratio 1:2	3%	5%	15%
MPL	2.5%	3%	8%

**Footnot:** Values adapted from [70].

**Table 13:** Proton permeability.

Liposomes	MPL	Egg Lecithin	Remarks
	+ 5µM Val	+ 5µM Val	
Valinomycin	4.5 × 10^−6^ × cm sec^−1^	10^−5^-10^−4^ × cm sec^−1^	Proton permeability coefficient
+ Val	−	+	excess valinomycin
+ Palm	−	++	
+ FCCP	+++	+++	
	2.7 × 10^−5^ nmol H^+^ sec^−1^ cm^−2^	1.2 × 10^−4^ nmol H^+^ sec^−1^ cm^−2^	Proton permeability

**Footnote:** Val, valinomycin; Palm, palmitic acid; MPL, main phospholipid, FCCP, carbonylcyanide-4-(trifluoromethoxy) phenylhydrazone; values adapted from [72].

**Table 14:** Effect of valinomycin on proton pumping and proton efflux.

Addition	Concentration	Initial Pump Rate	Proton Efflux [ng H^+^ x min^−1^]	
			Calculated per mg MPL	Calculated per mg BR
**nil**		1.4	0.6	0.1
valinomycin	0.24 µM	41.6	1.2	0.15
Val + gramicidin	0.24 µM + 1 µM	15.8	0.05	0.008
Val + gramicidin	0.24 µM + 5 µM	6.2	0.02	0.003
Val + FCCP	0.24 µM + 1 µM	15.5	0.21	0.035
Val + FCCP	0.24 µM + 6 µM	11.6	0.58	0.1
Val + FCCP	0.24 µM + 15 µM	6.3	0.58	0.1

**Footnote:** BR, bacteriorhodopsin; FCCP, carbonylcyanide-4-(trifluoromethoxy) phenylhydrazone; initial pump rate: ng H^+^ (mg BR x min)^−1^; values adapted from [72].

**Table 15:** Co-reconstitution and light-driven ATP synthesis.

Lipid	Liposomes Size [nm]	nmol ATP (mg × min)^−1^	Detergent/Method	Reference
MPL	132	13.4	Octylglucoside det-dialys/ French press	[65,66,72]
SBL	52	77.1	Taurodeoxycholate (TDOC)	[77,78,79,80]
DOPC	86	40.5		
ML	123	2.2		
DMPC	120	0		

**Footnote:** MPL, main phospholipid from *Thermoplasma acidophilum*; SBL, soybean lecithin; DOPC, dioleoyl phosphatidylcholine; ML, Micrococcus luteus lipid extract; DMPC, dimyristoyl- phosphatidylcholine; TDOC, taurodeoxycholate; det-dialys., detergent dialysis according to [65].

## Patents

Freisleben: H.-J.; Antonopoulos, E.; Bakowsky, U.; Rothe, U. Tetraetherlipide und diese enthaltende Liposomen sowie deren Verwendung. [Tetraether lipids and liposomes which contain them, and their use], German/European Patent 19607722.2; PCT/EP97/01011, 1996.

Freisleben, H.-J.; Gropp, F.; Hartmann, K.; Antonopoulos, E.; Balakirev, M.; Balakireva, L. Tetraetherlipidderivate und Tetraetherlipidderivate enthaltende Liposomen und Lipidagglomerate sowie deren Verwendung [Derivatives of tetraether lipids and these containing liposomes and agglomerates and their application]. German/International Patents (P 197 365 92.2; PCT/EP98/05264), 1997.

## Abbreviations

AFM	atomic force microscope
ATP	adenosine-triphosphate
BLM	black lipid membrane
BR	bacteriorhodopsin
CF	carboxyfluorescein
DHMG	1,2-dihexadecyl-3-*O-ß*-D-maltosyl-*sn*-glycerol
DMPC	dimyristoylphosphatidylcholine
DMSO	dimethyl-sulfoxide
DOC	deoxycholate
DOPC	dioleoylphosphatidylcholine
DPGG	dipalmitylglucosylglycerol
DPhG	diphytanylglycerol
DPhGG	diphytanylglucosylglycerol
DPhPC	diphytanylphosphatidylcholine
DPPC	dipalmitoylphosphatidylcholine
DPPG	dipalmitoylphosphatidylglycerol
DSPC	distearoylphosphatidycholine
DSM	Deutsche Sammlung von Mikroorganismen
DSC	differential scanning calorimetry
DTA	differential thermoanalysis
EL	egg (yolk) lecithin
EM	electron microscopy
EPR/ESR	electron paramagnetic resonance/electron spin resonance
FCCP	carbonylcyanide-4-(trifluoromethoxy)phenylhydrazone
GDNT	glycerol-dialkyl-nonitol-tetraether
GL	glycolipid
LPS	lipopolysaccharide
MGL	main glycolipid
ML	Micrococcus luteus lipid extract
MPL	main glycophospholipid from *Thermoplasma acidophilum*
OD	optical density
Palm	palmitic acid
PBFI	potassium-binding (benzofuran) fluorescence indicator
RT	room temperature
SBL	soybean lecithin
SDS	sodium dodecyl-sulfate
TDOC	taurodeoxycholate
TEL	tetraether lipid
T.a.	Thermoplasma acidophilum
TPLF	total polar lipid fraction
TPLF	Ta total polar lipid fraction from T.a.
Val	valinomycin
